# The safe removal of a superior mediastinal foreign body by mediastinoscopy: a case report

**DOI:** 10.1186/s40792-022-01525-3

**Published:** 2022-09-22

**Authors:** Takashi Sakuma, Tatsuro Tamura, Mikio Nambara, Ken Gyobu, Mami Yoshii, Takahiro Toyokawa, Shigeru Lee, Hiroaki Tanaka, Kazuya Muguruma, Masakazu Yashiro, Masaichi Ohira, Kiyoshi Maeda

**Affiliations:** Department of Gastroenterological Surgery, Graduate School of Medicine, Osaka Metropolitan University, 1-5-7 Asahi-Machi, Abeno-Ku, Osaka, Japan

**Keywords:** Mediastinal foreign body, Mediastinoscopy, Pneumomediastinal pressure

## Abstract

**Background:**

Mediastinal foreign bodies might cause mediastinal organ injury or mediastinal abscess. The prompt removal surgery of mediastinal foreign bodies is needed to prevent those complications. We report a case in which a mediastinal foreign body was removed by video-mediastinoscopy.

**Case presentation:**

The patient, a 74-year-old man with a chief complaint of hoarseness, was referred to our department for surgical management of a wooden foreign body that had traumatically migrated into the superior mediastinum. During the surgery, the video-mediastinoscopy was introduced under the pneumomediastinal pressure. We could dissect the scar tissue and remove the azalea tree branch safely and carefully, without damaging the other mediastinal organs. He was discharged on postoperative day 5, with no complications.

**Conclusions:**

Video-mediastinoscopic approach under pneumomediastinal pressure is minimally invasive and could provide wide surgical view. Therefore, we consider it useful and effective for removal of foreign bodies in the mediastinum.

## Background

Mediastinal foreign bodies might cause mediastinal organ injury or mediastinal abscess [1]. Furthermore, severe inflammation and the complexity of the mediastinal anatomy might make it difficult to remove them [2]. Thus, the prompt removal of mediastinal foreign bodies should be needed to prevent those complications. However, there is no consensus on the surgical managements and the timing of surgery. We herein used video-mediastinoscopy under pneumomediastinal pressure for a mediastinal foreign body removal operation.

## Case presentation

A 74-year-old man was refered to our department for the surgical management of a superior mediastinal foreign body. In July 2018, he was pruning his garden on a stepladder. He accidentally fell from the ladder, and an azalea tree branch was lodged in the external sternocleidomastoid muscle of his his left neck. He was immediately transported to the other hospital, and underwent the removal operation on the same day. For a while after surgery, he had no symptoms, such as fever or difficulty in swallowing. However, after 4 months in November 2018, he gradually felt hoarseness and revisited the same hospital. As a result of precise examinations, he was strongly suspected of the tree branch remaining.

**Fig. 1:**
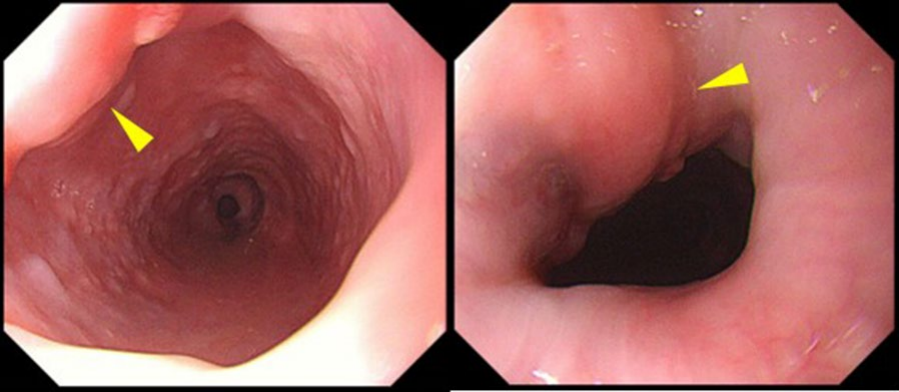
Preoperative upper gastrointestinal endoscopy. An elevated lesion was revealed on the anterior wall of the upper thoracic esophagus (arrowhead). This confirmed compression from something external

**Fig. 2:**
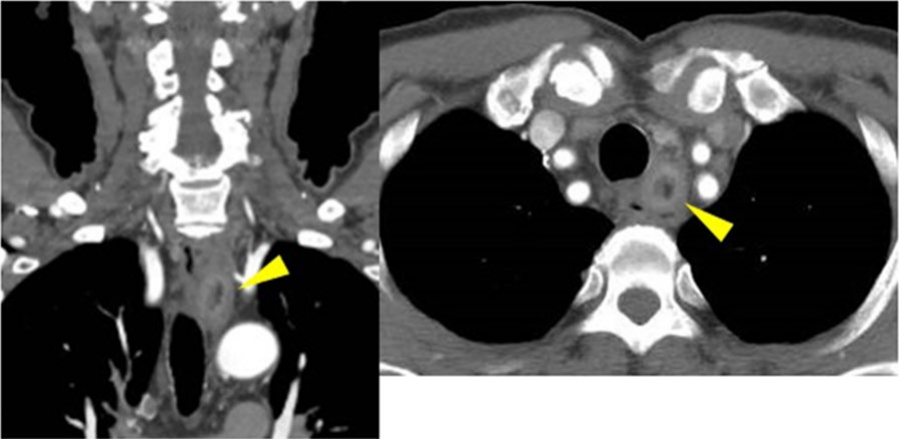
Contrast-enhanced CT. A superior mediastinal foreign body, located between trachea and esophagus, was sticking toward the internal, caudal side (arrowhead)

**Fig. 3:**
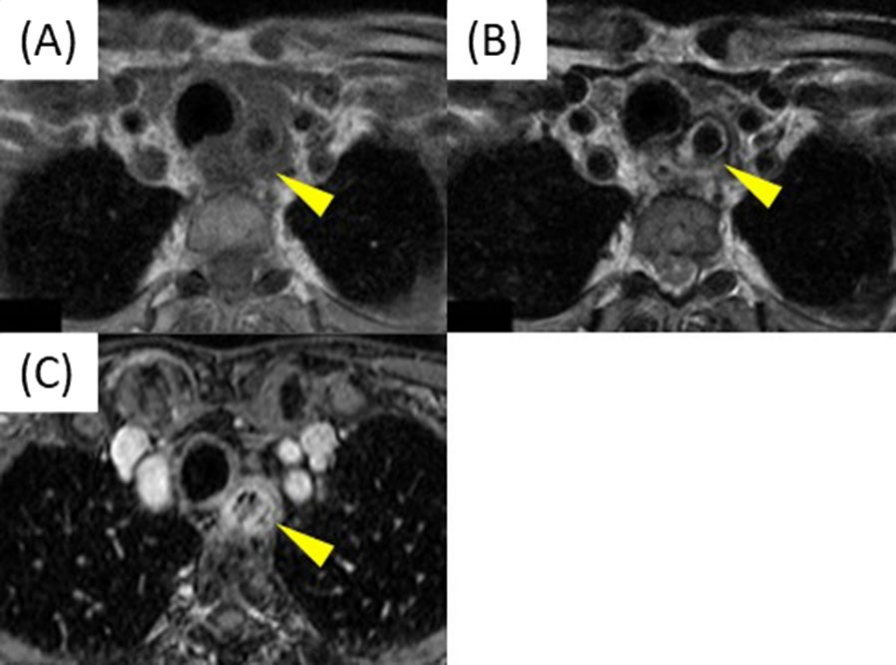
Contrast-enhanced MRI. Plain T1-weighted imaging (**A**), and plain T2 (**B**). Contrast-enhanced T1 (**C**) showed a non-signal line in the mediastinal foreign body

His physical examination and laboratory data revealed no remarkable findings. The left vocal cord was fixed in the paramedial position. Upper gastrointestinal endoscopy showed an elevated lesion on the anterior wall of the upper thoracic esophagus, 20 cm far from the incisors (Fig. 1). Contrast-enhanced computed tomography (CT) revealed a superior mediastinal linear foreign body with peripheral enhancement, located between trachea and esophagus (Fig. 2). The size was approximately 10 × 45 mm. There was no mediastinal abscess. To investigate the characteristics of the foreign body in detail, contrast-enhanced magnetic resonance imaging (MRI) was performed. The peripheral region revealed low intensity on both plain T1- and T2-weighted imaging, and on the other hand, the internal region revealed iso-intensity on plain T1-weighted imaging and high on plain T2 (Fig. 3A,B). And a non-signal line was revealed in the foreign body in contrast-enhanced T1-weighted imaging (Fig. 3C). As a result of the non-signal line and the MRI effect, we concluded that the foreign body was something wooden. He had the symptom of hoarseness and the risk of mediastinitis and esophagus perforation, so removal re-operation of the mediastinal foreign body was considered to be needed. Finally, in January 2019, we performed a foreign body removal re-operation by video-mediastinoscopy.

**Fig. 4:**
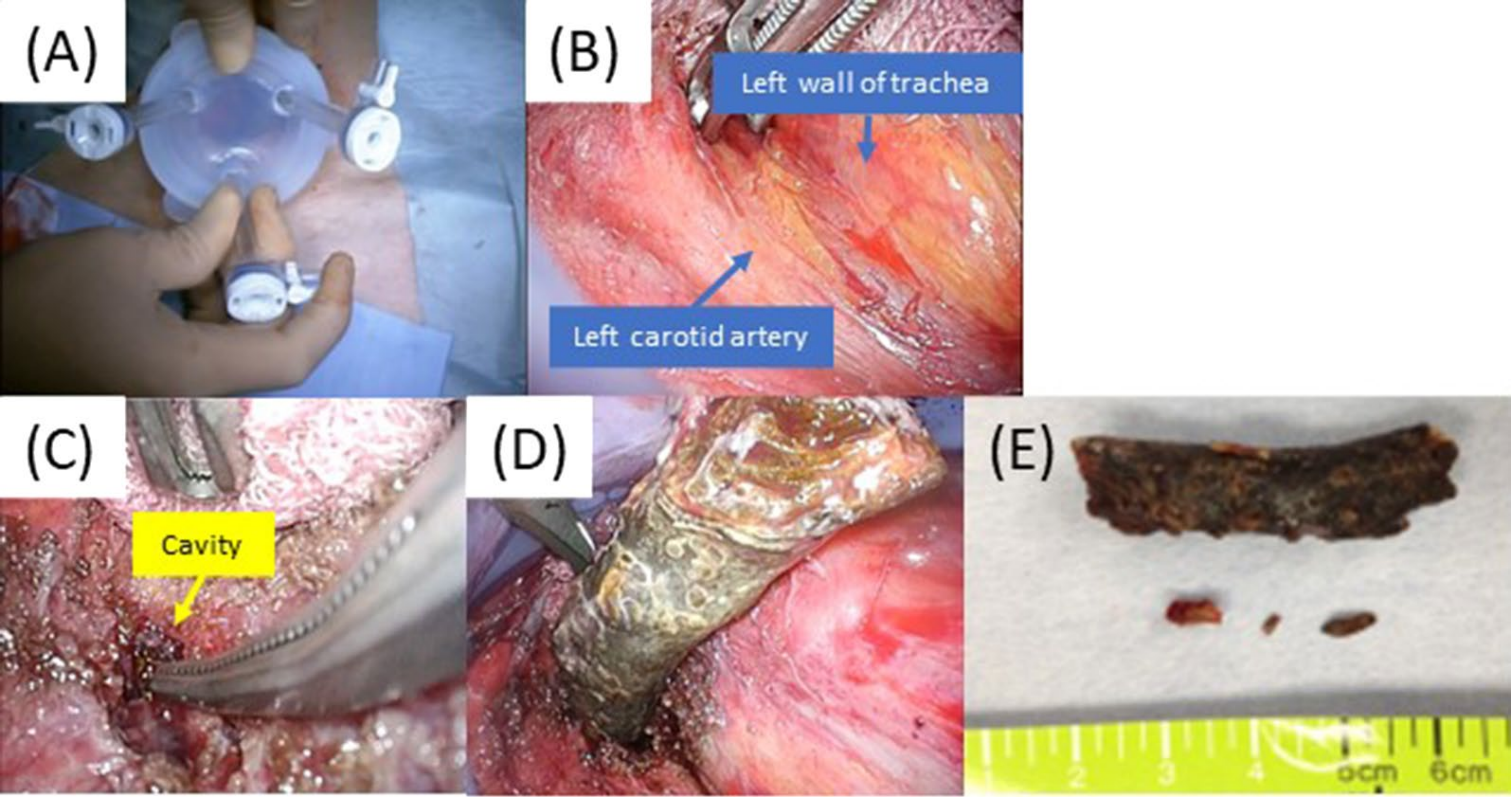
Intraoperative findings

Under general anesthesia, along the surgical scar on the left neck made in the first operation in July 2018, a 7-cm oblique incision was made. After exposing the inner edge of the sternocleidomastoid muscle, 70-mm diameter EZ-Access™ and Lap-Protector™ (Hakkou Shoji, Japan) were covered over the incision, and one 12-mm port and two 5-mm ports were inserted (Fig. 4A). Then, the video-mediastinoscopy was introduced through the 12-mm port, under pneumomediastinal pressure (5–8 cm H_2_O). With the wide surgical view, we dissected the scar tissue between the left wall of the trachea and the left common carotid artery to the caudal and dorsal sides (Fig. 4B). The scar tissue had become hard due to inflammation. Proceeding with the careful dissection, we reached into the cavity, where several twigs were removed. We further dissected the cavity to the dorsal side, and found a large wooden foreign body filling the whole cavity (Fig. 4C). We carefully performed adhesiolysis and gradually extended the cavity. Finally, an azalea tree branch, 4 cm long, was safely removed (Fig. 4D, E). After removal, upper gastrointestinal endoscopy was introduced during the surgery, and we confirmed no damage to the mucosa in the esophagus. We also investigated the cavity and confirmed neither foreign bodies remaining nor abscess. Left recurrent laryngeal nerve was not detected during the surgery. One drainage tube was placed at the bottom of the cavity, and the neck incision was closed. Operation time was 166 min, and blood loss volume was 45 ml.

**Fig. 5:**
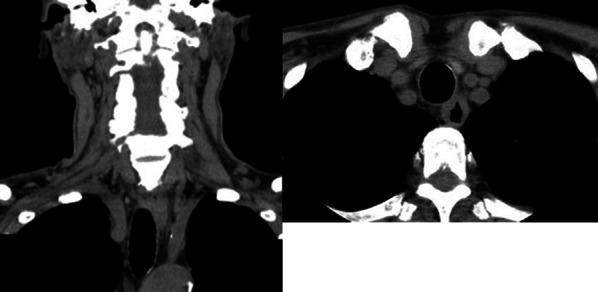
Postoperative CT. There were no remaining mediastinal foreign bodies

After the surgery, the patient was extubated without difficulty. Perioperative antibiotic therapy was discontinued on postoperative day 1. On postoperative day 2, the drainage tube was removed, and enteral feeding was initiated. The patient was discharged from our hospital on postoperative day 5, without any complications. Follow-up CT after the discharge revealed no mediastinal foreign bodies remaining (Fig. 5). Subsequent follow-up visits every 4 months demonstrated good wound healing, but he still felt hoarseness despite two surgeries, so he was taking vocalization rehabilitation.

## Discussion

Foreign bodies migrating into the mediastinum occur from direct injury or penetration by sharp foreign bodies in trachea and esophagus, for example, accidental ingestion or committing suicide. Mediastinal foreign bodies are associated with the considerable risk of descending necrotizing mediastinitis, mediastinal abscess, and the mediastinal organ injury, such as esophagus, trachea, or aorta [3]. Thus, the removal operation for them is considered to be required. However, multidisciplinary efforts of radiologists, gastroenterologists, anesthesiologists, and thoracic surgeons are needed for optimal preoperative planning, according to the shape, size and location of the mediastinal foreign bodies [2]. Furthermore, no consensus has been reached regarding surgical approach and the timing of surgery. But in our case, he had symptom of hoarseness and delayed treatment might lead esophagus perforation and mediastinitis, so he needed to undergo removal re-operation as prompt as possible.

A search on PubMed using the keywords “mediastinal foreign body” revealed 59 reported cases worldwide (iatrogenic migration cases were excluded). The cause of 13 cases (22.0%) is by direct injury. The types of foreign body have included fish bone (*n* = 8), needle (*n* = 7), a bullet (*n* = 7), teeth prosthesis (*n* = 5), and so on. Surgical removal approaches for them vary, and the most common approach is thoracotomy, 30 cases (50.8%). In recent years, thoracoscopic approaches have also increased. In any approach, to prevent damage to the mediastinal organs and foreign bodies remaining, we should remove them not by pulling out forcibly, but by dissecting the surrounding tissue carefully. Of 59 cases, there are only seven reported cases using video-mediastinoscopy (Table 1) [2, 4–9]. Four cases were caused due to direct injury by bullet, and three due to penetration into the mediastinum by accidental ingestion of pin stick, teeth prothesis, and fish bone. In all cases, the foreign bodies were located in the superior mediastinum. None of these reports described pneumomediastinal pressure.

**Table 1:** Characteristics of seven case reports and the present case

Author	Year	Cause of migration	Foreign body	Location	Pneumomediastinum pressure
Ward [4]	1970	Injury	Bullet	Superior	
Larrieu et al. [5]	1980	Injury	Bullet	Superior	
Faries et al. [6]	1996	Injury	Bullet	Superior	
Lynch et al. [7]	1999	Penetration	Pin stick	Superior	
Tekinbas et al. [8]	2007	Penetration	Teeth prothesis	Superior	
Metin et al. [9]	2013	Injury	Bullet	Superior	
Wang et al. [2]	2018	Penetration	Fish bone	Superior	
Our case	2019	Injury	Azalea tree branch	Superior	5–8 cm H_2_O

Mediastinoscopy has high diagnostic value and low rates of mortality and morbidity, resulting in mediastinoscopy as a widely used method for mediasitnal pathology, like preoperative nodal staging of lung cancer. [10, 11]. Recently it has been applied in esophageal cancer resection and cervical lymphadenectomy [12, 13]. In 1970, Ward [4] first reported the removal operation of a bullet migrating into the superior mediastinum by mediastinoscopy under local anesthesia. The mediastinum consists of several important organs, vessels, and nerves. Thus, highly meticulous procedures for mediastinal surgery are required. Video-mediastinoscopy and pneumomediastinal pressure could provide the expanded surgical view in narrow mediastinal region, and make the precise surgery. And more, since mediastinal approach does not need intrathoracic procedures, it could be safely introduced for the patients who have difficulty in thoracotomy due to severe respiratory failure or preoperatively suspected pleural adhesion. Compared to thoracotomy, mediastinoscopic surgery is more minimally invasive, and offers the advantage of reduced recovery time, reduced pain, and improved cosmesis [14]. In contrast, we must recognize the risk and complications of mediastinoscopic approach. The common complications include bleeding, pneumothorax, wound infection, tracheal injury, and recurrent nerve injury [15]. And when the mediastinoscopy is inserted, it could cause compression of the innominate artery, resulting in dissection or transient ischemic attack [16]. Furthermore, while we could obtain wide surgical view, our handlings are limited within a narrow retractor, so interference between surgeons could become the major problem. We must also pay attention to pneumomediastinal pressure. In transcervical minimally invasive esophagectomy of the animal model, from the perspective of restricted circulatory blood flow and lower perfusion thereafter, pressure over 12 mmHg will be inappropriate [17]. So, in summary, when vascular injury occurs and results in poor surgical view, it is necessary not to hesitate to introduce thoracotomy. And when bilateral recurrent laryngeal nerve injury occurs, it is important to prepare surgical airway on the patient. In our case, the patient had hoarseness, and considered the risk of postoperative respiratory failure, video-mediastinoscopic approach was preferred rather than the thoracoscopic approach.

The limitations of this single case report must be acknowledged; it is still unclear whether video-mediastinoscopy could be applied in all mediastinal foreign body cases. Therefore, it is necessary to consider the surgical indication of the mediastinoscopy according to the size and location of the foreign bodies. And more, of course, we could not prove the superiority of the video-mediastinoscopy over the thoracoscopy. Thus, further more cases are needed.

## Conclusions

This is the first reported case of the removal of mediastinal foreign bodies by video-mediastinoscopy under pneumomediastinal pressure. Our case suggests that it is minimally invasive and could provide expanded surgical view, so is recommended as one of the best surgical approaches for removal of mediastinal foreign bodies.

## Data Availability

The data supporting the conclusions of this article are included within the article.

## Abbreviations

CT: Computed tomography
MRI: Magnetic resonance imaging
